# Associations Between Gut Microbiota Abundance, Dietary Purine Intake, and Fecal Short-Chain Fatty Acids Across the Clinical Spectrum of Hyperuricemia: A Cross-Sectional Study in Mexican Adults

**DOI:** 10.3390/nu18203326

**Published:** 2026-10-10

**Authors:** Alim Caelli Fuentes-Bello, Karla Vanessa Guevara-Gonzalez, Omar Granados-Portillo, Laura Gabriela Sánchez-Lozada, Ricardo Jasso-Chávez, David Galeana-Cadena, Efrén Altamirano-Molina, Brígida Herrera-López, Laura Edith Martínez-Gómez, Carlos Martínez-Armenta, Luis Esaú López-Jácome, Regina Matesanz Sánchez Azcona, Mireya Martínez-García, Luis M. Amezcua-Guerra, Guadalupe Gutiérrez-Esparza, Carlos Pineda, Janitzia Vázquez-Mellado, Alberto G. López-Reyes, Gabriela Angélica Martínez-Nava

**Affiliations:** 1 Posgrado en Ciencias Biológicas, Universidad Nacional Autónoma de México, Unidad de Posgrado, Edificio D, 1° Piso, Circuito de Posgrados, Ciudad Universitaria, Coyoacán, Mexico City 04510, Mexico; caelli.fuentes@gmail.com; 2 Laboratorio de Gerociencias, Instituto Nacional de Rehabilitación Luis Guillermo Ibarra Ibarra, Calz México-Xochimilco 289, Coapa, Col. Arenal de Guadalupe, Tlalpan, Mexico City 14389, Mexico; karlavanessaguevara@gmail.com (K.V.G.-G.); david.microbiomics@gmail.com (D.G.-C.); vicky2017love@gmail.com (B.H.-L.); laurae.mtzg@gmail.com (L.E.M.-G.); c.armenta1208@gmail.com (C.M.-A.); allorey@yahoo.com (A.G.L.-R.); 3 Departamento de Fisiología de la Nutrición, Instituto Nacional de Ciencias, Médicas y Nutrición Salvador Zubirán, Mexico City 14080, Mexico; ograpo@yahoo.com; 4 Departamento de Fisiopatología Cardio-Renal, Instituto Nacional de Cardiología Ignacio Chávez, Juan Badiano 1, Belisario Domínguez Secc 16, Tlalpan, Mexico City 14080, Mexico; laura.sanchez@cardiologia.org.mx; 5 Departamento de Fisiología, Instituto Nacional de Cardiología Ignacio Chávez, Juan Badiano 1, Belisario Domínguez Secc 16, Tlalpan, Mexico City 14080, Mexico; rjassoch@gmail.com; 6 Universidad de la Salud (UNISA), Vasco de Quiroga 1345, Sta Fé, Álvaro Obregón, Mexico City 01210, Mexico; 7 Servicio de Reumatología, Instituto Nacional de Rehabilitación Luis Guillermo Ibarra Ibarra, Calz México-Xochimilco 289, Coapa, Col. Arenal de Guadalupe, Tlalpan, Mexico City 14389, Mexico; dragonblak41@gmail.com; 8 Laboratorio de Microbiología Clínica, Instituto Nacional de Rehabilitación Luis Guillermo Ibarra Ibarra, Calz México-Xochimilco 289, Coapa, Col. Arenal de Guadalupe, Tlalpan, Mexico City 14389, Mexico; esaulopezjacome@gmail.com; 9 Facultad de Ciencias de la Salud, Universidad Anáhuac Campus Sur, Av. de los Tanques 865, Torres de Potrero, Álvaro Obregón, Mexico City 01840, Mexico; matesanzsaregina@gmail.com; 10 Dental Public Health Department, Division of Graduate Studies and Research, School of Dentistry, Univesidad Nacional Autónoma de México, Mexico City 04510, Mexico; mireya.martinez@fo.unam.mx; 11 Departamento de Inmunología, Instituto Nacional de Cardiología Ignacio Chávez, Juan Badiano 1, Belisario Domínguez Secc 16, Tlalpan, Mexico City 14080, Mexico; lmamezcuag@gmail.com; 12 Investigadora por México, Secretaría de Ciencia, Humanidades, Tecnología e Innovación, Avenida Insurgentes Sur 1582, Crédito Constructor, Mexico City 03940, Mexico; gues.02@gmail.com; 13 Subdirección de Investigación, Instituto Nacional de Cardiología Ignacio Chávez, Juan Badiano 1, Belisario Domínguez Secc 16, Tlalpan, Mexico City 14080, Mexico; 14 Dirección General, Instituto Nacional de Rehabilitación Luis Guillermo Ibarra Ibarra, Calz México-Xochimilco 289, Coapa, Col. Arenal de Guadalupe, Tlalpan, Mexico City 14389, Mexico; carpineda@yahoo.com; 15 Servicio de Reumatología, Hospital General de México Eduardo Liceaga, Dr. Balmis 148, Doctores, Cuauhtémoc, Mexico City 06720, Mexico; jvazquezmellado@gmail.com

**Keywords:** gut microbiota, short-chain fatty acids, gout, hyperuricemia, dietary purines, *Prevotella*, *Akkermansia*, acetate, fecal metabolites

## Abstract

Background: Hyperuricemia and gout are closely associated with alterations in gut microbiota function. Short-chain fatty acids (SCFAs) produced by intestinal microorganisms may modulate immune responses and inflammation relevant to gout pathophysiology. This study evaluated fecal SCFA concentrations, their associations with dietary purine intake, and the relationship between *Akkermansia* spp. and *Prevotella* spp. abundance and fecal SCFA profiles across the clinical spectrum of hyperuricemia. Methods: This cross-sectional study included 380 Mexican adults: normouricemic controls (*n* = 141), individuals with asymptomatic hyperuricemia (AH; *n* = 98), and patients with gout (*n* = 141). Fecal SCFAs were quantified by GC-FID and bacterial abundance by qPCR. Dietary intake was assessed by semi-quantitative food frequency questionnaire and classified by purine content. Between-group differences were evaluated by the Kruskal–Wallis test (Benjamini–Hochberg correction); multivariable linear regression adjusted for age, sex, and BMI was used to assess associations, including a pre-specified dietary purine intake per clinical group interaction term. Results: Fecal acetate and propionate were significantly higher in gout patients than controls, whereas AH individuals had significantly higher butyrate. Moderate-purine food intake showed opposing associations with fecal acetate in controls (β = +3.61, *p* = 0.019) and gout patients (β = −4.59, *p* = 0.036), with a significant interaction (*p* = 0.004). Exploratory analyses revealed a positive association between *Prevotella* spp. abundance and fecal acetate exclusively in the gout group (β = 0.085, *p* = 0.020). Conclusion: These findings suggest distinct fecal SCFA profiles across stages of urate-related disease, with a disease-specific gut metabolic response to dietary purine intake potentially contributing to the inflammatory environment of established gout. Longitudinal studies with whole-microbiome profiling are needed to establish causality.

## 1. Introduction

Altered purine metabolism and reduced urinary urate excretion promote sustained elevations in serum urate concentrations, favoring the precipitation and deposition of monosodium urate (MSU) crystals in tissues and joints; which is the central pathophysiological process in the development of gouty arthritis [1]. Approximately 20–33% of circulating urate derives from dietary purines, whereas the remainder is derived from endogenous sources; high cellular turnover and reduced renal or intestinal excretion further contribute to its systemic accumulation [2]. Under physiological conditions, approximately two-thirds of urate is eliminated via the kidneys and the remaining is excreted through the gastrointestinal tract [3,4].

Asymptomatic hyperuricemia (AH) is defined as a persistently elevated urate concentration above 6.8 mg/dL in the absence of clinical manifestations. Although 85–90% of individuals with AH remain asymptomatic throughout their lives [5], this condition represents an important risk factor not only for the development of gout, but also for cardiovascular, renal, and metabolic diseases [6,7]. While all patients with gout necessarily progressed through a prior stage of AH, only an estimated 18.83% of hyperuricemic individuals ultimately develop gouty arthritis [6], suggesting that additional modulators participate in the transition from AH to clinically manifest disease.

The etiology of gout is multifactorial, involving genetic predisposition, metabolic comorbidities, and modifiable determinants such as diet [1]. In recent years, the gut microbiota has emerged as a novel factor associated with this rheumatic disease, and the interaction between urate metabolism and the intestinal microbial ecosystem has become a particularly active area of investigation [1].

Several metagenomic studies have documented specific alterations in the gut microbiota associated with hyperuricemia and gout. A metagenomic meta-analysis reported an enrichment of the genus *Prevotella* spp. in patients with gout, a finding linked to reduced capacity for urate degradation and diminished production of short-chain fatty acids (SCFAs) [7,8]. More specifically, *Prevotella copri* has been found to be enriched in patients with gout compared to those with AH [9]. Other studies have reported higher *Prevotella* spp. abundance in both AH individuals and gout patients relative to healthy controls [10].

In contrast, the genus *Akkermansia* has shown an opposing pattern, characterized by reduced relative abundance. A decline in *Akkermansia* spp. has been documented in individuals with AH compared to healthy controls, with a further reduction observed in gout patients relative to those with AH [10]. Consistent with these findings, recent reviews have described reduced *Akkermansia* spp. abundance, alongside genera such as Bifidobacterium, as a distinguishing feature of the gout-associated microbiome [11]. Emerging evidence further suggests a potentially protective role for members of the *Akkermansia* genus, with higher abundance reported in Gout patients without tophi (subcutaneous deposits of monosodium urate crystals) compared to those with more advanced disease presentations [12].

Short-chain fatty acids (SCFAs), primarily acetate, propionate, and butyrate, are the principal metabolic products of microbial fermentation of dietary fiber and resistant starch in the distal colon. Beyond their role as energy substrates for colonocytes, SCFAs exert systemic immunomodulatory effects through activation of G-protein-coupled receptors (GPR41, GPR43) and inhibition of histone deacetylases, promoting anti-inflammatory responses and gut barrier integrity [13,14]. In the context of chronic inflammatory conditions such as gout, where NLRP3 inflammasome activation by MSU crystals drives recurrent inflammatory episodes, reduced SCFA production may contribute to a permissive inflammatory environment. However, the relationship between gut microbiota composition, dietary purine intake, and fecal SCFA concentrations across the clinical spectrum of hyperuricemia has not been systematically characterized.

Diet represents one of the most influential determinants of the taxonomic and functional composition of the gut microbiota. Dietary patterns modulate the availability of metabolic substrates that selectively promote microbial community growth, impacting key pathways including polysaccharide fermentation, vitamin synthesis, and immune regulation [15,16]. Diets rich in dietary fiber promote the growth of SCFA-producing bacteria, while patterns high in saturated fat and simple sugars are associated with pro-inflammatory microbial profiles [17]. Notably, dietary changes can induce reproducible shifts in microbial composition within days [18], underscoring the central role of diet as a dynamic modulator of the host–microbiome relationship [19].

Mexico represents a population with a high and increasing burden of metabolic diseases, including obesity, diabetes, and metabolic syndrome, conditions closely associated with hyperuricemia and gout [20]. Furthermore, the Mexican diet is characterized by the coexistence of traditional dietary patterns alongside a marked increase in the consumption of ultra-processed products and sugar-sweetened beverages, a nutritional transition that may specifically modify gut microbiota composition and SCFA production [21]. Importantly, existing evidence linking gut microbiota alterations to hyperuricemia and gout derives predominantly from Asian and European cohorts [5,8,22,23], and population-specific evidence from Latin America remains scarce. Studying these relationships in a Mexican cohort may thus provide complementary insights into the microbiota–urate axis in a population with a distinct genetic background, metabolic risk profile, and dietary pattern.

In this context, the present study aimed to evaluate fecal SCFAs concentrations and their association with dietary purine intake across the clinical spectrum of hyperuricemia and gout in Mexican adults, and to explore whether the relative abundance of *Akkermansia* spp. and *Prevotella* spp. is associated with these functional metabolic profiles.

## 2. Materials and Methods

### 2.1. Study Design and Participants

This cross-sectional analysis was derived from a case-control study conducted at the Instituto Nacional de Rehabilitación Luis Guillermo Ibarra Ibarra (INR-LGII) in Mexico City, Mexico. The study protocol was approved by the institutional research and ethics review board of the INR-LGII (registration number: INRLGII 106/24) and conducted in accordance with the Declaration of Helsinki. Written informed consent was obtained from all participants prior to enrollment.

Three clinical groups were recruited: patients with confirmed gout (*n* = 141), individuals with AH (*n* = 98), and normouricemic healthy controls (*n* = 141). Gout was diagnosed according to the 2015 American College of Rheumatology/European League Against Rheumatism (ACR/EULAR) classification criteria [24]. AH was defined as at least two serum urate measurements exceeding 6.8 mg/dL in the absence of clinical manifestations of gout or urate crystal deposition disease. Controls were recruited as voluntary blood donors from the blood bank of the INR-LGII; eligibility required a serum urate concentration below 6.8 mg/dL at recruitment and no prior clinical history of hyperuricemia.

Participants were excluded if they reported use of antibiotics, antiparasitic agents, or short-chain fatty acid supplementation during the three months prior to fecal sample collection. Additional exclusion criteria included: active gastrointestinal disease, type 2 diabetes mellitus, metabolic syndrome, autoimmune diseases, chronic kidney disease, and history of kidney transplantation.

### 2.2. Clinical and Biochemical Assessment

Anthropometric measurements, including body weight, height, and waist circumference, were obtained using standardized procedures [25]. Body mass index (BMI) was calculated as weight in kilograms divided by height in meters squared. After an overnight fast of at least 8 h, venous blood samples were collected for biochemical analysis. Serum concentrations of uric acid, fasting glucose, total cholesterol, and triglycerides were measured by spectrophotometry of colorimetric enzymatic reactions following the manufacturer’s instructions (DiaSys, Holzheim, Germany) and using a microplate absorbance reader (iMark, Bio-Rad, Hercules, CA, USA).

### 2.3. Dietary Assessment

Dietary intake was assessed using a 58-item semi-quantitative food frequency questionnaire (FFQ) developed for this study. The instrument was based on a short FFQ originally validated in a Spanish adult population [25], which was selected for its practical length and coverage of key food groups relevant to purine intake classification. Food items were reviewed and culturally adapted for the Mexican population, including the addition of locally consumed foods such as corn and flour tortillas, traditional broths, and legume-based preparations. Predefined standardized serving sizes were included for each food item based on those specified in the original instrument, information from commercial food products available in Mexico, and consultation with a registered nutritionist. Participants reported the frequency of consumption for each food item over the preceding year using 12 standardized response categories ranging from never (0) to daily (7). Weekly intake equivalents were calculated from reported frequencies, and the proportional weekly intake of each food group was estimated as the weekly frequency of that food category divided by the total weekly frequency of all reported food items. As this FFQ has not been formally validated in Mexican adults with hyperuricemia, dietary purine intake estimates derived from this instrument were considered exploratory.

Foods were classified by purine content according to the categorization proposed by Kaneko et al. [26]: low-purine foods containing less than 50 mg per 100 g (Category 1), moderate-purine foods containing 50–100 mg per 100 g (Category 2), and high-purine foods containing more than 100 mg per 100 g (Category 3). Category 1 included dairy products, most cereals and breads, tortillas, rice, eggs, most fruits and juices, oils, and cheeses. Category 2 included fresh salads, leafy vegetables (spinach, chard), pasta, soups and broths, and mixed preparations including Mexican antojitos and breaded dishes. Category 3 included all meats (poultry, red meat, and processed meats), all fish and seafood, and beer.

### 2.4. Fecal Sample Collection and Short-Chain Fatty Acid Quantification

Fecal samples were collected by participants at home on the same day as their scheduled visit for questionnaire administration. All participants were instructed to provide a fresh stool sample (the first bowel movement of the day). Prior to collection, participants received standardized instructions from trained personnel on the correct collection procedure and were provided with the necessary materials. All samples were transported at 4 °C and stored at −80 °C within two hours of its collection.

Fecal concentrations of acetate, propionate, and butyrate were quantified by gas chromatography following a previously described protocol with minor modifications [27]. Briefly, SCFAs were extracted from 150 mg of fecal sample with 0.1 N hydrochloric acid. Chromatographic separation was performed using an Agilent 6850 gas chromatograph equipped with a flame ionization detector and a DB-225MS capillary column (30 m × 0.25 mm internal diameter × 0.25 μm film thickness; J&W Scientific, Santa Clara, CA, USA). One microliter of the sample extract was injected in split mode at a ratio of 1:20.8 at an injector temperature of 225 °C. Hydrogen was used as the carrier gas at a constant flow rate of 0.5 mL/min, and the interface temperature was maintained at 225 °C. Quantification was performed against external calibration curves constructed from certified standards of acetic, propionic, and butyric acids, and peak areas were obtained from the generated chromatograms. Concentrations are expressed in mmol.

### 2.5. Fecal DNA Extraction and Gut Microbiota Quantification

Total genomic DNA was extracted from fecal samples using the QIAamp DNA Stool Kit (QIAGEN, Hilden, Germany) according to the manufacturer’s instructions. The relative abundance of *Akkermansia* spp. and *Prevotella* spp. was determined by real-time quantitative PCR (qPCR) using genus-specific primers and the 16S rRNA gene as the endogenous reference for normalization.

These genera were selected a priori based on published evidence linking their abundance to SCFA production, gut barrier function, and inflammatory states relevant to gout and hyperuricemia. Critically, our group has previously documented differential abundance of both *Akkermansia* spp. and *Prevotella* spp. across the same three clinical groups (normouricemic controls, individuals with AH, and patients with gout) in Mexican adults from the same institution: Akkermansia showed higher abundance in gout patients compared to AH individuals, and Prevotella was significantly more abundant in both AH and gout patients compared to controls [10]. *Akkermansia muciniphila* has been associated with metabolic health and intestinal permeability, while *Prevotella* spp. have been implicated in joint inflammation in rheumatic diseases and in acetate production in the context of hyperuricemia. These observations in the Mexican population, together with broader evidence from international cohorts, support the biological relevance of these genera as targeted candidates for SCFA-related analyses in this context.

Primer sequences and amplification conditions are detailed in Supplementary Table S1. Briefly, *Akkermansia*-specific primers [28] were amplified under the following cycling conditions: initial denaturation at 95 °C for 2 min, followed by 30 cycles of 95 °C for 15 s, 63 °C for 15 s, and 72 °C for 60 s. *Prevotella*-specific primers [22] and universal 16S rRNA primers were amplified under the following conditions: initial denaturation at 95 °C for 10 min, followed by 40 cycles (16S rRNA gene: 30 cycles) of 95 °C for 15 s and 60 °C for 45 s. Each 10 μL reaction contained 5.0 μL Maxima SYBR Green master mix (Thermo Scientific, Waltham, MA, USA), 1.0 μL each of forward and reverse primers at 10 μM (0.25 μL each for *Prevotella* spp.), 2.0 μL nuclease-free water (3.5 μL for *Prevotella* spp.), and 1.0 μL template DNA. All reactions were performed on a Rotor-Gene Q instrument (QIAGEN, Hilden, Germany). Reaction efficiencies were monitored per tube and confirmed to be comparable across all three primer sets. Relative abundance was calculated using the 2^−ΔCt^ method, where ΔCt represents the difference between the Ct value of the target genus and the Ct value of the universal 16S rRNA reference gene.

### 2.6. Statistical Analysis

Data normality was assessed using the Shapiro–Wilk test. Continuous variables are presented as means ± standard deviations (SD) for normally distributed data, and as medians with interquartile ranges (IQR) for non-normally distributed data. Categorical variables are presented as frequencies and percentages. Between-group differences in continuous variables were evaluated using one-way ANOVA or the Kruskal–Wallis test, as appropriate, with Dunn’s post-hoc test with Benjamini–Hochberg correction for multiple comparisons. Chi-squared tests were used for categorical variables.

Prior to regression analyses, continuous variables were transformed to meet normality assumptions. Fecal acetate and butyrate concentrations were square root-transformed; propionate was log-transformed. Relative abundances of *Akkermansia* spp. *and Prevotella* spp. were log-transformed. All transformations were verified by visual inspection of Q-Q plots and residual distributions.

Multivariable linear regression models were used to evaluate the following associations: (1) relative bacterial abundance and fecal SCFA concentrations, and (2) dietary purine intake and fecal SCFA concentrations. All models were adjusted for age, sex, and BMI and stratified by clinical group. The association between moderate-purine food intake (Category 2) and fecal acetate concentration was further evaluated in a pooled model including a multiplicative interaction term (Category 2 intake × clinical group indicator), with normouricemic controls as the reference category. The clinical group was coded using two indicator variables representing gout and AH, respectively.

Two pre-specified sensitivity analyses were conducted. First, all primary regression models were repeated in the male-only subgroup, given the markedly imbalanced sex distribution across clinical groups, particularly the near-exclusive male composition of the gout group (98.6% male), and the known uricosuric effect of estrogens, which renders premenopausal women biologically less susceptible to hyperuricemia. Women with AH in the present sample were all postmenopausal, representing a hormonal context distinct from that of premenopausal controls. Second, analyses were restricted to participants within the overlapping range of age (35–60 years) and BMI (22–36 kg/m^2^) across all three groups, representing the region of common support. Results of both sensitivity analyses are reported in Supplementary Tables S3 and S4.

A hierarchical analytical approach was adopted to address the risk of false-positive findings arising from multiple comparisons. Pairwise post-hoc comparisons following Kruskal–Wallis tests were corrected using the Benjamini–Hochberg (BH) false discovery rate method. The interaction analysis between moderate-purine food intake and clinical group on fecal acetate concentration was designated as the primary regression analysis, based on the study’s primary objective. Stratified diet–SCFA and bacteria–SCFA regression models were conducted as secondary and exploratory analyses, respectively, and are interpreted as hypothesis-generating. No correction for multiple testing was applied across the set of regression models, consistent with standard practice in observational nutritional epidemiology for a limited set of biologically motivated comparisons [23,29].

Due to gout patients were not naive to urate-lowering therapy (ULT) and could be under treatment at the time of fecal sample collection, two complementary analyses were conducted to evaluate the potential influence of pharmacological treatment on the observed associations: (1) fecal acetate concentrations were compared between patients with and without each medication class using the Mann–Whitney test; (2), the association between *Prevotella* spp. abundance and fecal acetate in the gout group was re-evaluated with sequential adjustment for use of urate-lowering therapy, colchicine, and glucocorticoids, separately and simultaneously. All analyses were performed using Stata version 19 (StataCorp, College Station, TX, USA). Statistical significance was defined as a two-sided *p*-value < 0.05.

## 3. Results

### 3.1. Study Population Characteristics

A total of 380 participants were included: 141 normouricemic controls, 141 patients with gout, and 98 individuals with AH. The clinical and biochemical characteristics of the study population are presented in Table 1. The three groups differed significantly in age, with patients with gout being older than controls (median 54 vs. 45 years, respectively). Urate concentrations differed significantly across groups, as expected by their clinical definitions. The sex distribution was markedly imbalanced: the gout group was composed almost exclusively of men (98.6%), while controls and individuals with AH included a higher proportion of women (29% and 14.3%, respectively). Women with AH in this sample were all postmenopausal. Regarding pharmacological treatment, 88.7% of gout patients were receiving ULT (allopurinol 81.6%, febuxostat 7.1%), 51.1% colchicine, and 7.8% glucocorticoids at the time of fecal sample collection.

### 3.2. Fecal Short-Chain Fatty Acid Amounts Across Clinical Groups

Fecal amounts of the three SCFAs measured differed significantly across clinical groups (Table 2). For acetate, the Kruskal–Wallis test indicated significant between-group differences (*p* < 0.001). Post-hoc Dunn’s analysis with Benjamini–Hochberg correction revealed that gout patients had significantly higher acetate than both normouricemic controls (*p* < 0.001) and individuals with AH (*p* = 0.004). Individuals with AH also had significantly higher acetate than controls (*p* = 0.045).

For butyrate, overall between-group differences were significant (*p* = 0.045). Individuals with AH had significantly higher butyrate than normouricemic controls (*p* = 0.021). No significant differences were observed between gout patients and controls (*p* = 0.214) nor between gout patients and individuals with AH (*p* = 0.090).

On the other hand, propionate differed significantly between-groups (*p* = 0.018). Both gout patients and individuals with AH showed significantly higher propionate than normouricemic controls (*p* = 0.012 and *p* = 0.021, respectively). Propionate amount did not differ significantly between gout patients and individuals with AH (*p* = 0.496).

### 3.3. Dietary Purine Intake and Fecal Acetate: Interaction Analysis

Given that the adapted FFQ was not formally re-validated in the Mexican population (see Limitations), all dietary findings should be interpreted as exploratory. The proportional weekly intake of food groups classified by purine content across clinical groups is presented in Table 3. Low-purine foods (Category 1, <50 mg/100 g) constituted the predominant dietary component in all groups, accounting for approximately 74–76% of total weekly food intake. Moderate-purine foods (Category 2, 50–100 mg/100 g) represented 11.3% (IQR 6.0%) in controls, 12.7% (IQR 7.6%) in gout patients, and 13.2% (IQR 6.8%) in individuals with AH. High-purine foods (Category 3, >100 mg/100 g) contributed approximately 11% of the diet in all three groups. Notably, no statistically significant between-group differences were observed in the proportional intake of any purine category: low-purine foods (*p* = 0.167), moderate-purine foods (*p* = 0.250), or high-purine foods (*p* = 0.477), indicating comparable dietary purine profiles across clinical groups at the time of assessment.

**Figure 1:**
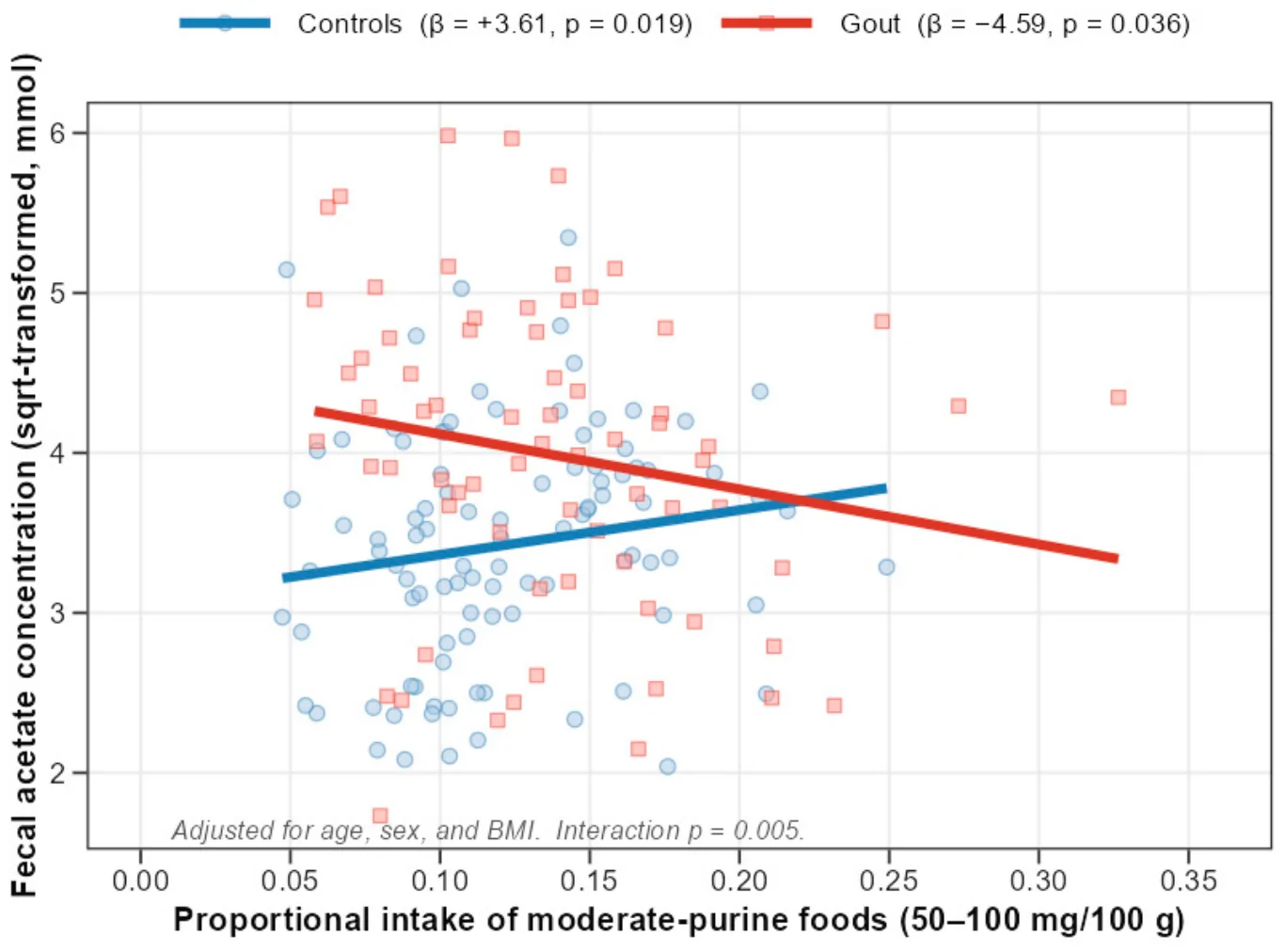
Stratified regression lines depicting the association between the proportional intake of moderate-purine foods (50–100 mg/100 g) and square root-transformed fecal acetate concentrations in normouricemic controls and patients with gout. Blue circles correspond to controls’ and red squares to gout patients’ values. Lines represent observed regression slopes from adjusted models. In controls, higher moderate-purine food intake was positively associated with fecal acetate (β = +3.61, *p* = 0.019), whereas in gout patients the association was negative (β = −4.59, *p* = 0.036). The multiplicative interaction term was statistically significant in the pooled adjusted model (β = −6.97, *p* = 0.005). Note: dietary analyses in the gout group are considered exploratory given the prevalent case design.

The association between the proportional intake of moderate-purine foods (50–100 mg/100 g, per Kaneko et al. [26]) and fecal acetate was evaluated using stratified adjusted regression models and a pooled interaction model. In stratified analyses, moderate-purine food intake was positively associated with fecal acetate in normouricemic controls (β = +3.61, 95% CI 0.60–6.62, *p* = 0.019) and negatively associated in patients with gout (β = −4.59, 95% CI −8.88 to −0.29, *p* = 0.036). No significant association was observed in individuals with AH (β = −0.051). These opposing directional patterns are illustrated in Figure 1.

In the pooled interaction model adjusted for sex, age, and BMI, the multiplicative interaction term between moderate-purine food intake and gout group membership was statistically significant (β = −6.97, 95% CI −11.85 to −2.08, *p* = 0.005), confirming that the association between dietary purine intake and fecal acetate differs significantly between gout patients and normouricemic controls. The interaction term for AH did not reach statistical significance (β = −3.76, *p* = 0.136). Sex was an independent predictor of fecal acetate in the pooled model (β = +0.55, *p* < 0.001). Because patients with gout in this sample are prevalent cases who may have modified their dietary habits following diagnosis, these findings should be interpreted as exploratory and require confirmation in prospective studies or incident case designs. These lines are confusing should be rewritten.

### 3.4. Relative Abundance of Akkermansia spp. and Prevotella spp. Across Clinical Groups

**Figure 2:**
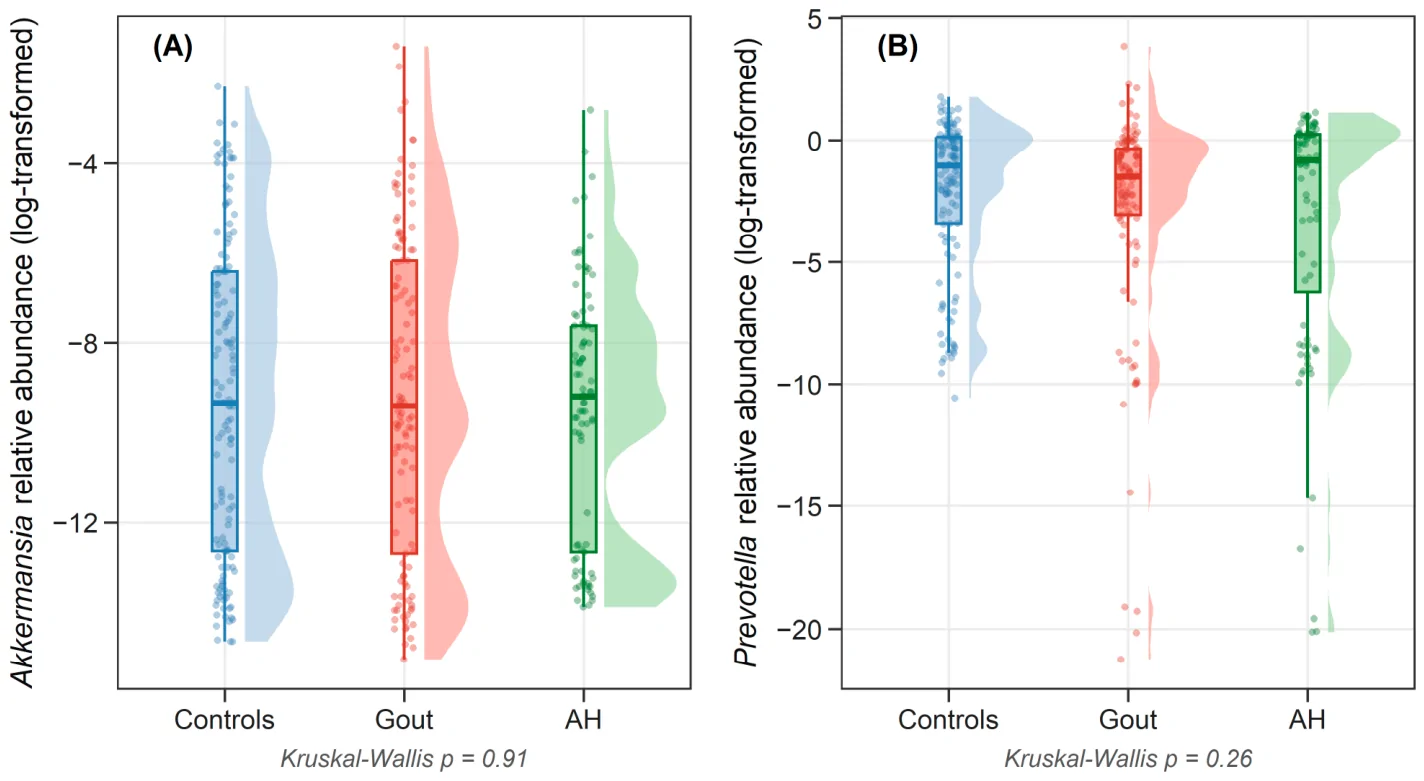
Relative abundance of *Akkermansia* spp. (**A**) and *Prevotella* spp. (**B**) across clinical groups. Relative abundance was quantified by real-time qPCR and expressed using the 2^−ΔCt^ method. No significant differences were observed between groups (Kruskal–Wallis: *p* = 0.93 for *Akkermansia* spp.; *p* = 0.28 for *Prevotella* spp.). AH: asymptomatic hyperuricemia.

The relative abundance of *Akkermansia* spp. and *Prevotella* spp. quantified by qPCR and expressed using the 2^−ΔCt^ method, did not differ significantly across clinical groups. Median (IQR) log-transformed relative abundance of *Akkermansia* spp. was −9.33 (6.21) in controls, −9.30 (6.51) in gout patients, and −9.17 (5.44) in individuals with AH (Kruskal–Wallis *p* = 0.93). Similarly, no significant differences were observed for *Prevotella* spp. with median values of −1.04 (3.57), −1.49 (2.70), and −0.87 (7.42) in controls, gout, and AH groups, respectively (Kruskal–Wallis *p* = 0.28). These findings are illustrated in Figure 2. Although no significant differences in abundance were detected, these results should be interpreted in the context of the targeted quantification approach used, which was designed to test specific a priori hypotheses regarding these two genera rather than to characterize the broader taxonomic composition of the gut microbiota.

### 3.5. Associations Between Bacterial Abundance and Fecal Short-Chain Fatty Acid Concentrations

Multivariable linear regression models adjusted for age and BMI were used to evaluate the associations between the relative abundance of *Akkermansia* spp. and *Prevotella* spp. and fecal SCFA amounts, stratified by clinical group. Complete data for these models were available for a subset of participants due to missing fecal SCFA or bacterial abundance values (*n* = 263 for SCFA analyses; *n* = 203 for combined SCFA and microbiota analyses); available sample sizes are reported for each model.

No significant associations were observed between *Akkermansia* spp. abundance and fecal acetate, propionate, or butyrate in any of the three clinical groups after adjustment (all *p* > 0.05; Supplementary Table S2).

**Figure 3:**
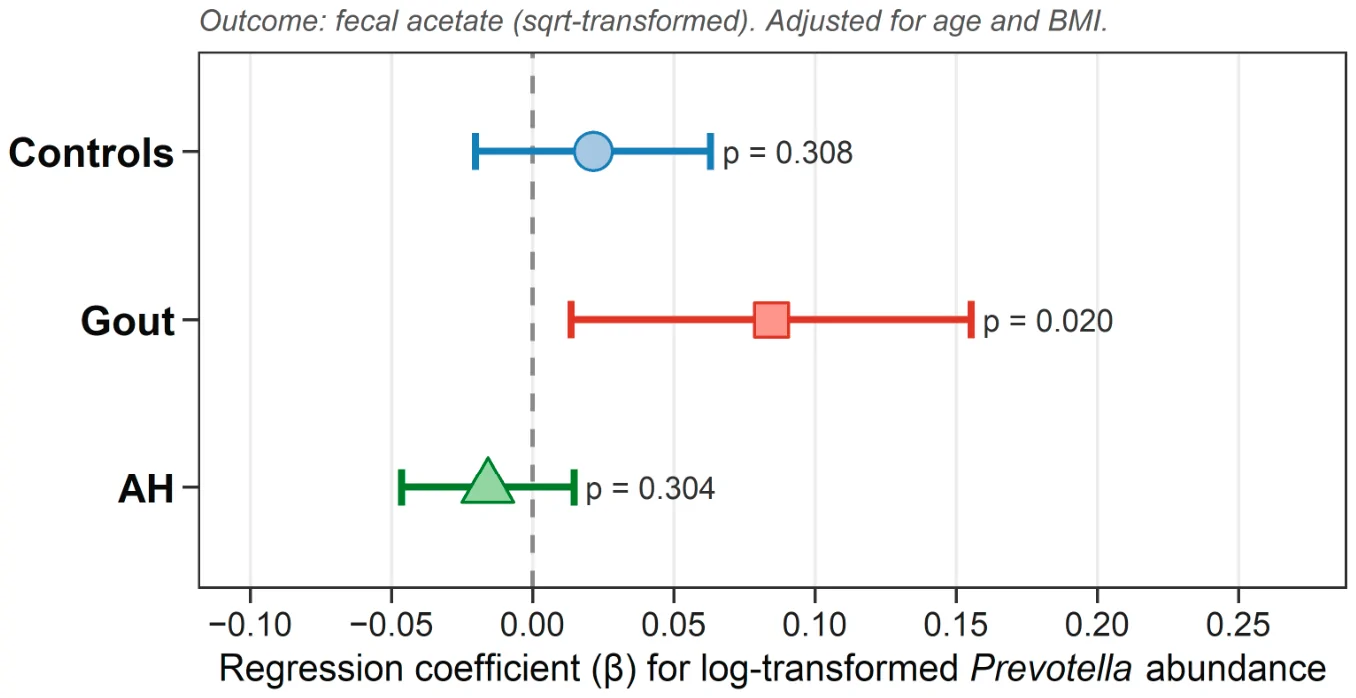
Coefficient plot showing regression coefficients (β) with 95% confidence intervals for the association between *Prevotella* spp. relative abundance and fecal acetate concentrations across clinical groups, derived from adjusted multivariable linear regression models. The dashed vertical line indicates the null value (β = 0). The association was statistically significant only in the gout group. AH: asymptomatic hyperuricemia.

For *Prevotella* spp. a significant positive association with fecal acetate was observed exclusively in patients with gout (β = 0.085, 95% CI 0.014–0.155, *p* = 0.020, *n* = 51), independent of BMI and age. This association was not significant in normouricemic controls (β = 0.020, *p* = 0.348, *n* = 84) or in individuals with AH (β = −0.016, *p* = 0.338, *n* = 64). The group-specific nature of this finding is illustrated in Figure 3. No significant associations were observed between *Prevotella* spp. abundance and butyrate or propionate in any clinical group after adjustment (all *p* > 0.05; Supplementary Table S2).

### 3.6. Sensitivity Analyses

Three pre-specified sensitivity analyses were conducted. The first two are reported in full in Supplementary Tables S3 and S4; the third is reported in Supplementary Table S5.

When analyses were restricted to men only (*n* = 327: 100 controls, 139 gout, 89 AH), the association between *Prevotella* spp. abundance and fecal acetate in gout patients remained unchanged (β = 0.084, *p* = 0.020), as the gout group was already composed almost exclusively of men. The interaction term between moderate-purine food intake and gout did not reach conventional significance in the male-only model (β = −4.65, *p* = 0.093), suggesting that the significant interaction observed in the full sample may be partially influenced by the sex imbalance across groups and should be interpreted with caution.

When analyses were restricted to participants within the overlapping range of age (35–60 years) and BMI (22–36 kg/m^2^) across all three groups (n = 273: 116 controls, 83 gout, 74 AH), the association between *Prevotella* spp. abundance and fecal acetate in gout patients was stronger than in the primary analysis (β = 0.107, 95% CI 0.039–0.176, *p* = 0.003), and the interaction term between moderate-purine food intake and gout remained statistically significant (β = −6.90, *p* = 0.013). Notably, the interaction term between moderate-purine food intake and AH group membership also reached statistical significance in the common support model (β = −5.50, 95% CI −10.87 to −0.13, *p* = 0.045), a finding not observed in the primary analysis (*p* = 0.063), suggesting that this association may be attenuated by the heterogeneity in age and BMI across groups. These results indicate that the primary findings are robust to demographic heterogeneity across groups, and that the marginal association observed for the AH group in the primary analysis may reflect partial attenuation by differences in age and BMI distribution rather than a true absence of effect. Although propensity-score matching or inverse probability weighting could provide additional control for confounding by these variables, the present common-support analysis offers a transparent and interpretable approach to assessing the robustness of the primary findings within a comparable demographic range.

A third sensitivity analysis evaluated the potential influence of pharmacological treatment on fecal acetate concentrations in the gout group. Univariate comparisons indicated higher fecal acetate in patients receiving ULT (median [IQR]: 4.22 [3.64–4.77] vs. 3.32 [2.33–3.95], *p* = 0.016) and colchicine (4.47 [3.66–4.96] vs. 3.92 [3.20–4.30], *p* = 0.014) compared to patients not receiving these medications; no significant difference was observed for glucocorticoids (*p* = 0.109; Supplementary Table S5, Part A). However, the reference group for ULT was small (*n* = 7), limiting the interpretation of this comparison. In multivariate models additionally adjusted for each pharmacological covariate, the association between *Prevotella* spp. abundance and fecal acetate remained across all models (Model 1: β = 0.086, 95% CI 0.014–0.157, *p* = 0.019; Model 2: β = 0.081, 95% CI 0.010–0.151, *p* = 0.026; Model 3: β = 0.080, 95% CI 0.009–0.151, *p* = 0.028). No pharmacological covariate reached statistical significance in any model (all *p* ≥ 0.21); however, the wide confidence intervals for urate-lowering therapy (β = 0.337, 95% CI −0.454 to 1.128 in Model 1) and glucocorticoids (β = 0.558, 95% CI −0.351 to 1.467 in Model 3) reflect the small untreated reference group (*n* = 7) and indicate insufficient statistical power to rule out pharmacological confounding; a type II error in these comparisons cannot be excluded (Supplementary Table S5, Part B).

## 4. Discussion

The present study characterized fecal SCFA profiles across the clinical spectrum of hyperuricemia and identified distinct patterns associated with clinical status and dietary purine intake. The main findings can be summarized in three major points. First, gout patients exhibited significantly higher fecal acetate concentrations compared to normouricemic controls, whereas individuals with AH showed significantly higher butyrate than controls. Fecal propionate was significantly higher in both gout patients and AH individuals compared with normouricemic controls, while no significant difference was observed between AH and gout patients. This differential fecal SCFA profile is consistent with distinct gut metabolic environments across the clinical spectrum of hyperuricemia; however, whether this pattern reflects broader metabolic alterations (including unmeasured metabolites such as succinate, secondary bile acids, or branched-chain fatty acids) cannot be established from the present data and will require untargeted metabolomic profiling in future studies. Second, the proportional intake of moderate-purine foods showed opposing associations with fecal acetate in controls and gout patients, with a statistically significant interaction that survived adjustment for age, sex, and BMI. Given the central role of diet in shaping microbial fermentation and SCFA production [30], these interaction findings raise the hypothesis that the gut microbiota of gout patients may respond differently to dietary purine substrates than that of normouricemic individuals; however, this hypothesis requires confirmation in longitudinal prospective studies with controlled dietary assessment and comprehensive microbiome profiling. Finally, in an exploratory analysis, the relative abundance of *Prevotella* spp. was positively associated with fecal acetate in the gout group, a finding not observed in controls or individuals with AH.

The elevated fecal amount of acetate in gout patients observed in the present study are consistent with prior metabolomic characterization of fecal samples from individuals with gout. A metabolomic study of fecal and serum samples from Chinese patients found significantly higher concentrations of acetate alongside glycine, taurine, and succinate compared to healthy controls [31]. Additionally, functional predictions from 16S rRNA gene sequencing have similarly identified a higher abundance of acetate-producing bacteria in the gut microbiota of gout patients compared to individuals with AH [7,10,32]. Taken altogether with the present findings, these observations point to a consistent pattern of elevated acetate production in the intestinal environment of gout patients, which may reflect both compositional and functional alterations of the gut microbiota in this context.

In contrast to the acetate findings in gout, individuals with AH exhibited significantly higher fecal butyrate compared to normouricemic controls. This finding is consistent with functional predictions from 16S rRNA sequencing indicating a higher abundance of butyrate- and propionate-producing bacteria in the gut microbiota of individuals with AH compared to gout patients [10]. Butyrate is a well-characterized anti-inflammatory SCFA that suppresses NLRP3 inflammasome activation through inhibition of histone deacetylases and activation of GPR109a, thereby limiting IL-1β production [33,34]. In the context of hyperuricemia, persistent NLRP3 activation by circulating urate is a key driver of the inflammatory amplification that culminates in acute gout [1]. The relatively elevated butyrate production in individuals with AH may therefore represent a microbiota-mediated contribution to an intestinal anti-inflammatory environment that contributes to maintaining the subclinical state despite persistent hyperuricemia. This hypothesis is mechanistically grounded in the work of Vieira et al. [35] and Macia et al. [36], who demonstrated, respectively, that GPR43-mediated acetate signaling potentiates the response to MSU crystals and that GPR109a-mediated butyrate signaling suppresses NLRP3 inflammasome activation, and is consistent with the observation that only a minority of hyperuricemic individuals ultimately develop clinical gout [6]. However, as with all cross-sectional observations, the direction of causality cannot be established; it remains possible that the butyrate differences reflect dietary or other lifestyle factors that differ between groups rather than a protective microbiota-mediated mechanism. Collectively, these observations suggest a model in which the gut microbial metabolic environment may differentially modulate the inflammatory threshold in AH and gout through SCFA-mediated receptor signaling: elevated butyrate in AH may support an anti-inflammatory intestinal environment through GPR109a-mediated suppression of NLRP3 activation, whereas the elevated acetate observed in gout patients may have opposing effects.

The differential association between moderate-purine food intake and fecal acetate across clinical groups constitutes a novel finding that has not been previously reported. To our knowledge, no prior study has examined the relationship between dietary purine classification and fecal SCFA concentrations in individuals with AH or gout. Importantly, the absence of significant between-group differences in the proportional intake of moderate-purine foods indicates that the observed interaction is unlikely to reflect differential dietary purine exposure across groups. Rather, these data suggest that comparable dietary purine loads elicit distinct metabolic responses in the gut depending on the clinical context, a finding that implicates the intestinal environment associated with gout, rather than dietary behavior per se, as the modulating factor. The positive association in normouricemic controls (higher moderate-purine intake correlated with higher acetate) and the negative association in gout patients (higher intake correlated with lower acetate), confirmed by a statistically significant interaction term, suggest that the gut metabolic response to the same dietary stimulus differs according to the urate-related clinical context. This could reflect differences in microbial community composition, intestinal transit, substrate availability, or the broader alterations in intestinal physiology associated with the chronic inflammatory state of established gout. Importantly, the interaction term between moderate-purine food intake and AH group membership only reached statistical significance when analyses were restricted to the region of demographic overlap across groups (common-support model: β = −5.50, 95% CI −10.87 to −0.13, *p* = 0.045), compared to the primary analysis (*p* = 0.063). This suggests that a portion of the attenuation observed in the full analytic sample may reflect the wider heterogeneity in age and BMI distribution across clinical groups, rather than a true biological absence of effect in AH individuals. Accordingly, the interpretive implications of the observed interaction estimates should account for the possibility that demographic distributional differences, not solely disease-specific biological mechanisms, contribute to the magnitude and pattern of the associations reported.

In addition, the intestine plays a central role in urate homeostasis, accounting for approximately one-third of total urate excretion through extra-renal elimination. Impaired intestinal urate excretion has been recognized as a common mechanism contributing to hyperuricemia, and alterations in the gut microbiota may influence this process by modifying the abundance or activity of uricolytic microorganisms, intestinal barrier function, and the local metabolic environment. Therefore, the altered acetate response observed in gout patients may reflect not only differences in microbial fermentation but also broader alterations in intestinal physiology linked to urate handling. Although these mechanisms were not directly evaluated in the present study, they provide a plausible biological framework linking gut microbial metabolism with urate homeostasis and warrant further investigation.

The exploratory association between *Prevotella* spp. abundance and fecal acetate, observed exclusively in the gout group, provides a potential microbial link to the elevated amount of acetate in this population. *Prevotella* spp. produces acetate primarily through the fermentation of complex carbohydrates and proteins, and its enrichment in patients with gout and inflammatory conditions has been documented in metagenomic studies [10,11]. The genus-specificity and group-specificity of this association is consistent with the notion that the relationship between microbial composition and metabolite output may be modified by the intestinal environment associated with established gout. This finding is particularly relevant in light of experimental evidence demonstrating that acetate potentiates the inflammatory response to monosodium urate (MSU) crystals in germfree murine models through GPR43-mediated signaling, amplifying neutrophil recruitment and IL-1β production [35]. The preferential association between *Prevotella* spp. abundance and fecal acetate in the gout group, absent in controls and in individuals with AH, raises the hypothesis that *Prevotella*-derived acetate may contribute to the pro-inflammatory intestinal environment characteristic of established gout, potentially lowering the threshold for MSU crystal-induced NLRP3 inflammasome activation. However, the small effective sample size for this model and the absence of whole-microbiome profiling limit the interpretation of this finding, which should be considered hypothesis-generating. Direct testing of this hypothesis will require prospective studies with measurement of GPR43 signaling, fecal SCFA quantification, and intestinal immune activation in individuals across the clinical spectrum of hyperuricemia.

The absence of significant differences in *Akkermansia* spp. or *Prevotella* spp. abundance across clinical groups is noteworthy in the context of studies reporting lower *Akkermansia* spp. and altered *Prevotella* spp. in gout or AH individuals [10,11]. This discrepancy may reflect methodological differences: the present study used targeted qPCR quantification of two specific genera, which provides high analytical precision but does not capture the full ecological context of the microbiota. Changes in *Akkermansia* spp. or *Prevotella* spp. in other studies were detected primarily through 16S rRNA amplicon sequencing of the whole microbial community, where compositional shifts may be more apparent due to relative abundance dynamics across the entire ecosystem. Additionally, the Mexican adult population studied here may differ from the predominantly Asian cohorts in which microbiota alterations in gout have been most extensively characterized, highlighting the need for population-specific investigations [37].

The high prevalence of pharmacological treatment in the gout group raised the question of whether the elevated fecal acetate observed in this group could reflect a pharmacological effect rather than disease-related alterations in gut microbial metabolism. Univariate comparisons indicated higher fecal acetate concentrations in patients receiving ULT and colchicine compared to untreated patients. However, these differences were not independent in multivariate models: after adjustment for age, BMI, and *Prevotella* spp. abundance, neither ULT nor colchicine was significantly associated with fecal acetate (all *p* ≥ 0.21), and the *Prevotella* spp.–acetate association remained consistent across all pharmacological sensitivity models. The univariate associations likely reflect confounding by disease severity, since patients with more severe gout are both more likely to require combination pharmacotherapy and may have a more profoundly altered intestinal environment. Together, these results suggest that the elevated fecal acetate concentrations observed in gout patients are not primarily attributable to pharmacological treatment, although the small reference group size (*n* = 7 without ULT) precludes definitive conclusions about isolated pharmacological effects on fecal SCFA production. Additionally, pharmacological treatment in the gout group, particularly colchicine, which alters intestinal motility, could theoretically modify the fermentative response to dietary purine intake; this possibility cannot be excluded from the present data and warrants evaluation in future studies with larger untreated gout cohorts.

The present study has several limitations that should be considered. First, the prevalent case design in the gout group is particularly constraining for dietary analyses, as it cannot be determined whether the observed diet–SCFA associations reflect pre-existing patterns or post-diagnosis behavioral modifications. Second, the moderate-purine food category employed in this study is heterogeneous in terms of fermentation potential, encompassing both fermentable vegetables (spinach, fresh salads) and mixed processed preparations (croquettes, antojitos); this heterogeneity limits the mechanistic attribution of the observed diet–SCFA associations to purine content specifically, and future studies should employ more granular dietary assessment instruments to address this. Third, the food frequency questionnaire used in this study was adapted from a validated instrument originally developed for the general Hispanic adult population [38] and was not formally re-validated in the Mexican population or in individuals with hyperuricemia or gout. Although food items were culturally adapted and serving sizes standardized in consultation with a registered nutritionist, the absence of formal re-validation represents a limitation of the dietary assessment component. No validated purine-specific dietary assessment instrument currently exists for Mexican or Latin American populations, which constrained instrument selection. Future studies should prioritize the development and validation of culture-specific dietary tools for individuals with hyperuricemia. Fourth, the targeted qPCR approach captures only two bacterial genera, limiting the characterization of the broader microbial functional context. The moderate-purine food category is heterogeneous in terms of fermentation potential, making it difficult to attribute the observed diet–SCFA associations to purine content specifically. Fifth, disease duration data were available only for participants with gout and not for the AH group, owing to the clinically silent nature of AH, in which no identifiable clinical event marks disease onset. Accordingly, we were unable to adjust for disease duration in the main regression models, and the potential influence of gout chronicity as an independent modifier of gut microbiota composition and fecal SCFA concentrations cannot be fully excluded. Additionally, the cross-sectional design of this study precludes the establishment of temporal relationships and does not allow determination of whether the observed gut microbial and metabolic differences preceded or resulted from disease onset. Future studies integrating longitudinal sampling and comprehensive microbiome profiling are needed to characterize the directionality and dynamics of these associations. The robustness of the primary Prevotella–acetate association to adjustment for pharmacological treatment provides partial reassurance that disease-management effects are not the primary driver of the observed findings. Finally, the marked sex imbalance across groups (98.6% male in gout) means that findings in the gout group should be interpreted as specific to men with established gout and cannot be extrapolated to women.

Despite these limitations, the present study contributes several elements to the understanding of gut metabolic function in the context of hyperuricemia. It provides the first direct quantification of fecal SCFAs across three defined clinical groups representing the spectrum from normouricemia through AH to established gout in a Mexican adult population. The differential SCFA profiles, with higher butyrate in AH and higher acetate in gout, generate a testable hypothesis about microbiota-mediated metabolic environments that may contribute to disease progression or protection, which can be addressed in longitudinal studies with incident gout cases. Furthermore, the interaction between dietary purine intake and clinical status on fecal acetate represents a novel dietary–microbiome association warranting further investigation with more granular dietary assessment methods and prospective designs.

## 5. Conclusions

Patients with gout and individuals with AH exhibited distinct fecal SCFA profiles compared to normouricemic controls, with gout characterized by higher acetate and propionate and AH by higher butyrate. Moderate-purine food intake showed opposing associations with fecal acetate in controls and gout patients, with a statistically significant interaction. Although the targeted quantification of *Akkermansia* spp. and *Prevotella* spp. did not reveal abundance differences across groups, *Prevotella* spp. abundance was positively associated with fecal acetate in gout patients in exploratory analyses. The primary associations were robust to adjustment for pharmacological treatment as neither ULT nor colchicine was independently associated with fecal acetate in multivariate models. Together, these findings are consistent with a model in which the gut microbiota contributes to distinct intestinal metabolic environments across the clinical spectrum of hyperuricemia, potentially influencing the transition from AH to clinically manifest gout. Longitudinal studies with incident gout cases and whole-microbiome profiling are needed to establish causality and to characterize the mechanisms underlying these associations.

## Figures and Tables

**Table 1:** Clinical and biochemical characteristics of the study population by clinical group.

Variable	Controls (*n* = 141)	Gout (*n* = 141)	AH (*n* = 98)	*p*-Value
Age (years)	45 (12) ^a^	54 (17) ^b^	48 (14) ^a^	<0.001 ^†^
Male sex, n (%)	100 (70.90)	139 (98.60)	84 (85.70)	<0.001 ^‡^
BMI (kg/m^2^)	26.30 (3.89) ^a^	28.74 (5.12) ^b^	28.93 (4.5) ^b^	<0.001 ^†^
Urate (mg/dL)	5.60 (1.23) ^a^	6.79 (2.89) ^b^	7.60 (0.83) ^c^	<0.001 ^†^
Fasting glucose (mg/dL)	94.34 (18.39) ^a^	98.18 (14.26) ^b^	98.80 (16.20) ^b^	<0.001 ^†^
Triglycerides (mg/dL)	114.38 (50.94) ^a^	162.82 (99.50) ^b^	133.60 (80.95) ^c^	<0.001 ^†^
Total cholesterol (mg/dL)	161.87 (70.00) ^a^	171.17 (52.06) ^b^	188.52 (53.18) ^c^	<0.001 ^†^
Disease duration (years)	—	8 (2–16)	—	—
Any urate lowering therapy (*n,*%)	NA	125 (88.65%)	NA	—
Allopurinol (*n*,%)	NA	115 (81.56%)	NA	—
Febuxostat (*n*,%)	NA	10 (7.09%)	NA	—
Colchicine (*n*,%)	NA	72 (51.1%)	NA	—
Glucocorticoids (*n*,%)	NA	11 (7.8%)	NA	—

Data expressed as median (interquartile range) or *n* (%), as appropriate. Pharmacological treatment was assessed at the time of fecal sample collection and is reported for the gout group only, as urate-lowering therapy and gout-specific medications are not indicated in normouricemic controls or individuals with AH. ^†^ *p*-value from Kruskal–Wallis test; ^‡^ *p*-value from chi-squared test. Post-hoc pairwise comparisons (Dunn’s test, Benjamini–Hochberg correction): Values sharing the same superscript letter do not differ significantly (*p* ≥ 0.05, Dunn’s test with Benjamini–Hochberg correction); different letters indicate statistically significant pairwise differences (*p* < 0.05). AH: asymptomatic hyperuricemia; BMI: body mass index.; NA: Not applicable.

**Table 2:** Fecal short-chain fatty acid amounts across clinical groups.

Variable	Controls (*n* = 141)	Gout (*n* = 141)	AH (*n* = 98)	*p*-Value
Acetate	12.03 (6.41) ^a^	16.53 (11.06) ^b^	13.55 (5.40) ^c^	<0.001 ^†^
Propionate	3.56 (1.78) ^a^	4.31 (3.32) ^b^	4.00 (2.28) ^b^	0.018 ^†^
Butyrate	2.72 (1.63) ^a^	3.14 (3.03) ^ab^	3.35 (2.26) ^b^	0.045 ^†^

Data expressed as median (interquartile range) in mmol. ^†^ *p*-value from Kruskal–Wallis test with Dunn’s post-hoc test (Benjamini–Hochberg correction). Pairwise adjusted *p*-values: acetate (gout vs. controls *p* < 0.001, AH vs. controls *p* = 0.026, gout vs. AH *p* = 0.005); propionate (gout vs. controls *p* = 0.012, AH vs. controls *p* = 0.021); butyrate (AH vs. controls *p* = 0.021); Superscript letters indicate post-hoc groupings: different letters denote significant pairwise differences (*p* < 0.05). Statistical analyses were performed on square root-transformed (acetate, butyrate) and log-transformed (propionate) values; descriptive statistics are reported in original units. AH: asymptomatic hyperuricemia.

**Table 3:** Proportional weekly dietary intake of food groups classified by purine content, by clinical group.

Dietary Category (Purine Content)	Controls (*n* = 139)	Gout (*n* = 128)	AH (*n* = 98)	*p*-Value
Low-purine foods (<50 mg/100 g)	76.1 (9.7)	74.3 (9.5)	75.0 (9.2)	0.167
Moderate-purine foods (50–100 mg/100 g)	11.3 (6.0)	12.7 (7.6)	13.0 (6.7)	0.250
High-purine foods (>100 mg/100 g)	11.5 (5.7)	11.7 (6.9)	11.3 (5.8)	0.477

Data are expressed as the percentage of total reported weekly food-consumption frequency represented by each food category. Food classification based on Kaneko et al. [26]. *p*-values from Kruskal–Wallis test. AH: asymptomatic hyperuricemia.

## Data Availability

The data supporting the findings of this study are not publicly available due to privacy and ethical restrictions governing the use of clinical data collected at the Instituto Nacional de Rehabilitación ‘Luis Guillermo Ibarra Ibarra’. De-identified data may be made available upon reasonable request to the corresponding author, subject to institutional approval.

## Supplementary Materials

The following supporting information can be downloaded at https://www.mdpi.com/article/10.3390/nu18203326/s1; Table S1: Primer sequences and real-time qPCR amplification conditions for quantification of *Akkermansia* spp. and *Prevotella* spp. relative abundance in fecal samples; Table S2: Associations between gut microbiota abundance and fecal short-chain fatty acid concentrations by clinical group; Table S3: Sensitivity analysis restricted to male participants; Table S4: Sensitivity analysis restricted to participants within the common support region (age 35–60 years and BMI 22–36 kg/m^2^; *n* = 273); Table S5: Pharmacological treatment in gout patients and sensitivity analyses.

## Abbreviations

The following abbreviations are used in this manuscript:

AH	Asymptomatic Hyperuricemia
BMI	Body mass index
CI	Confidence interval
INR-LGII	Instituto Nacional de Rehabilitación ‘Luis Guillermo Ibarra Ibarra’
IQR	Interquartile range
MSU	Monosodium urate
qPCR	Quantitative polymerase chain reaction
rRNA	Ribosomal Ribonucleic acid
SCFA	Short chain fatty acids
ULT	Urate-lowering therapy
