# Supplementary material for: Associations Between Gut Microbiota Abundance, Dietary Purine Intake, and Fecal Short-Chain Fatty Acids Across the Clinical Spectrum of Hyperuricemia: A Cross-Sectional Study in Mexican Adults
Source: Nutrients. 2026 Oct 10;18(20):3326. doi: 10.3390/nu18203326 (PMC13708603; doi:10.3390/nu18203326)
Supplement: Supplementary file 1 [file nutrients-18-03326-s001.zip › nutrients-4554880-supplementary.pdf]

Table S1. Primer sequences and real-time qPCR amplification conditions for quantification of *Akkermansia* spp. and *Prevotella* spp. relative abundance in fecal samples.

Target	Primer sequences (5'→3')	Amplification conditions	Reaction mix (per 10 µL reaction)
**16S rRNA** (reference gene)	**Forward:** 5'-AAACTCAAAKGAATTGACGG-3'<br>**Reverse:** 5'-CTCACRRCACGAGCTGAC-3'	95°C — 10 min 30 cycles:<br>95°C — 15 s<br>60°C — 45 s	SYBR Green master mix: 5.0 µL<br>Forward primer (10 µM): 1.0 µL<br>Reverse primer (10 µM): 1.0 µL<br>Nuclease-free H_2O: 2.0 µL<br>Template DNA: 1.0 µL
Akkermansia spp.(Pai et al., 2022)	**Forward:** 5'-CAGCACGTGAAGGTGGGGAC-3'<br>**Reverse:** 5'-CCTTGCGGTTGGCTTCAGAT-3'	95°C — 2 min<br>30 cycles:<br>95°C — 15 s<br>63°C — 15 s<br>72°C — 60 s	SYBR Green master mix: 5.0 µL<br>Forward primer (10 µM): 1.0 µL<br>Reverse primer (10 µM): 1.0 µL<br>Nuclease-free H_2O: 2.0 µL<br>Template DNA: 1.0 µL
Prevotella spp.(Elnaggar et al., 2023)	**Forward:** 5'-AGCATTAAGCATCCCACC-3'<br>**Reverse:** 5'-ACTTAAGCCGACACCTCAC-3'	95°C — 10 min<br>40 cycles:<br>95°C — 15 s<br>60°C — 45 s	SYBR Green master mix: 5.0 µL<br>Forward primer (10 µM): 0.25 µL<br>Reverse primer (10 µM): 0.25 µL<br>Nuclease-free H_2O: 3.5 µL<br>Template DNA: 1.0 µL

SYBR Green master mix: Maxima SYBR Green qPCR Master Mix (Thermo Scientific, CA, USA) or equivalent. All reactions performed on Rotor-Gene Q (QIAGEN, Germany). K = G or T (IUPAC degenerate base code); R = A or G.

Table S2. Associations between gut microbiota abundance and fecal short-chain fatty acid concentrations by clinical group.

Outcome (SCFA)	Controls (n=85)	Gout (n=51)	AH (n=67)
**A. *Akkermansia* spp. relative abundance**			
**Acetate** (sqrt-transformed)	0.001 (−0.040, 0.041) p = 0.969	0.018 (−0.045, 0.082) p = 0.564	−0.005 (−0.070, 0.039) p = 0.567
**Butyrate** (sqrt-transformed)	0.016 (−0.008, 0.041) p = 0.179	−0.019 (−0.060, 0.022) p = 0.347	−0.031 (−0.067, 0.005) p = 0.087
**Propionate** (log-transformed)	−0.004 (−0.029, 0.021) p = 0.727	0.001 (−0.039, 0.041) p = 0.977	0.006 (−0.037, 0.027) p = 0.762
**B. *Prevotella* spp. relative abundance**			
**Acetate** (sqrt-transformed)[†]	0.021 (−0.020, 0.063) p = 0.308	0.085 (0.014, 0.155) p = 0.020[*]	−0.015 (−0.040, 0.024) p = 0.612
**Butyrate** (sqrt-transformed)	−0.018 (−0.043, 0.007) p = 0.161	−0.005 (−0.054, 0.043) p = 0.829	−0.007 (−0.028, 0.014) p = 0.505
**Propionate** (log-transformed)	0.011 (−0.013, 0.036) p = 0.358	0.041 (−0.003, 0.086) p = 0.069	0.017 (−0.004, 0.034) p = 0.112

[*] $p < 0.05$. Statistically significant association. All models adjusted for age and BMI (sex omitted in the gout group due to collinearity) Analyses restricted to participants with complete fecal SCFA and microbiota data (n = 203: controls n = 85, gout n = 51, AH n = 67), representing a subset of the full study cohort (N = 380).CI: confidence interval; β: regression coefficient for log-transformed bacterial relative abundance. SCFA: short-chain fatty acid; AH: asymptomatic hyperuricemia. Sqrt: square root.

Table S3. Sensitivity analysis restricted to male participants.

Variable / Model	Controls (n = 100)	Gout (n = 139)	AH (n = 89)
**Part A.** *Prevotella* spp. relative abundance and fecal acetate (adjusted for age and BMI; men only)			
β (95% CI)	0.026 (−0.020, 0.072)	0.084 (0.014, 0.155)[*]	−0.009 (−0.042, 0.025)
p-value	0.258	0.020	0.607
adj. R^2	0.037	0.167	−0.026
**Part B**. Interaction model: moderate-purine intake × group and fecal acetate (n =200; adjusted for age and BMI; men only)			
**Term**	**β**	**95% CI**	**p-value**
Gout vs. controls	0.994	(0.245, 1.743)	0.010
AH vs. controls	0.369	(−0.420, 1.157)	0.358
Moderate-purine intake (controls ref.)	0.984	(−3.313, 5.281)	0.652
Purines × gout	−4.654	(−10.133, 0.824)	0.093
Purines × AH	−2.397	(−8.168, 3.374)	0.414
Age	−0.003	(−0.014, 0.007)	0.502
BMI	−0.018	(−0.040, 0.010)	0.162
**Part C.** Stratified analysis: moderate-purine intake and fecal acetate (adjusted for age and BMI; men only)			

Variable / Model	Controls (n = 100)	Gout (n = 139)	AH (n = 89)
**Metric**	**Controls**	**Gout**	**AH**
β (95% CI)	0.889 (−2.673, 4.450)	−4.586 (−8.872, −0.300)[*]	−0.561 (−3.560, 2.438)
p-value	0.619	0.036	0.710

[*] $p < 0.05$. Part A models adjusted for age and BMI (sex omitted, as the sample is restricted to men). Parts B and C adjusted for age and BMI. AH: asymptomatic hyperuricemia; CI: confidence interval; β: regression coefficient.

Table S4. Sensitivity analysis restricted to participants within the common support region (age 35–60 years and BMI 22–36 kg/m²; n = 273).

Variable / Model	Controls (n = 116)	Gout (n = 83)	AH (n = 74)
**Part A.** *Prevotella* spp., relative abundance and fecal acetate (adjusted for age, sex, and BMI)			
β (95% CI)	0.023 (−0.030, 0.077)	0.107 (0.039, 0.176)[*]	0.016 (−0.026, 0.058)
p-value	0.388	0.003	0.445
adj. R^2	−0.032	0.308	−0.010
**Part B.** Interaction model: moderate-purine intake × group → fecal acetate (adjusted for age, sex, and BMI)			
**Term**	**β**	**95% CI**	**p-value**
Gout vs. controls	1.214	(0.443, 1.989)	0.002
AH vs. controls	0.761	(0.006, 1.515)	0.048
Moderate-purine intake (controls ref.)	3.274	(−0.428, 6.977)	0.083
**Purines × Gout**[*]	−6.947	(−12.438, −1.457)	0.013
Purines × AH	−5.502	(−10.875, −0.130)	0.045
Sex (male)	0.724	(0.441, 1.008)	<0.001
Age	−0.007	(−0.022, 0.008)	0.388
BMI	−0.014	(−0.048, 0.021)	0.435

[*] $p < 0.05$. Part A models adjusted for age and BMI; Part B adjusted for age, sex, and BMI. AH: asymptomatic hyperuricemia

Table S5. Pharmacological treatment in gout patients and sensitivity analyses.

Medication	Without treatment Median (IQR)	With treatment Median (IQR)	p-value
**Part A.** Univariate comparison of fecal acetate concentrations by pharmacological treatment status within the gout group.			
**Any urate-lowering therapy**	3.32 (2.33–3.95)[b]	4.22 (3.64–4.77)	0.016[*]
**Colchicine**	3.92 (3.20–4.30)	4.47 (3.66–4.96)	0.014[*]
**Glucocorticoids**	4.04 (3.32–4.59)	4.77 (4.50–4.78)	0.109

Part A. Univariate comparison of fecal acetate concentrations by pharmacological treatment status within the gout group.

Medication	Without treatment<br>Median (IQR)	With treatment<br>Median (IQR)	p-value

Part B. Multivariate sensitivity analyses: association between *Prevotella* spp. abundance and fecal acetate in gout patients with sequential pharmacological adjustment.

Term	Model 1<br>(+ ULT)	Model 2<br>(+ Colchicine)	Model 3<br>(+ ULT + Colchicine + GC)
***Prevotella* spp. abundance (predictor of interest)**			
**β (95% CI)**	0.086 (0.014, 0.157)*	0.081 (0.010, 0.151)*	0.080 (0.009, 0.151)*
**p-value**	0.019	0.026	0.028
**Pharmacological covariates β (95% CI)**			
**Any urate-lowering therapy**	0.337 (−0.454, 1.128)	—	0.279 (−0.507, 1.065)
p-value	0.396		0.478
**Colchicine**	—	0.306 (−0.179, 0.790)	0.198 (−0.314, 0.709)
p-value		0.211	0.440
**Glucocorticoids**	—	—	0.558 (−0.351, 1.467)
p-value			0.222
**Other covariates**			
**BMI (kg/m²)**	−0.042 (−0.107, 0.023)	−0.046 (−0.107, 0.015)	−0.037 (−0.102, 0.028)
p-value	0.202	0.136	0.254
**Age (years)**	−0.018 (−0.041, 0.006)	−0.017 (−0.040, 0.006)	−0.010 (−0.035, 0.014)
p-value	0.134	0.154	0.402

[*] $p < 0.05$.

[b] Reference group size is small (n = 7 without ULT), limiting the reliability of this comparison.

The association between *Prevotella* abundance and fecal acetate in gout patients was consistent across all pharmacological sensitivity models (β range 0.080–0.086, all $p < 0.05$). No pharmacological covariate reached statistical significance in any multivariate model (all $p \geq 0.21$).

Taken together, the univariate associations observed in Part C (higher acetate with ULT and colchicine use) are not independent after multivariate adjustment, suggesting they reflect confounding by disease severity rather than a direct pharmacological effect on fecal acetate production.

ULT: urate-lowering therapy; GC: glucocorticoids; AH: asymptomatic hyperuricemia; CI: confidence interval; β: regression coefficient.
